# Chronic Hypoxia-Induced Microvessel Proliferation and Basal Membrane Degradation in the Bone Marrow of Rats Regulated through the IL-6/JAK2/STAT3/MMP-9 Pathway

**DOI:** 10.1155/2020/9204708

**Published:** 2020-01-23

**Authors:** Mingming Zhu, Min Yang, Quanyu Yang, Wenling Liu, Hui Geng, Li Pan, Lu Wang, Rili Ge, Linhua Ji, Sen Cui, Zhanquan Li

**Affiliations:** ^1^Research Center for High Altitude Medicine, Qinghai University, Xining 810001, China; ^2^Key Laboratory of Application and Foundation for High Altitude Medicine Research in Qinghai Province, Xining 810001, China; ^3^Affiliated Hospital of Qinghai University, Xining 810001, China

## Abstract

Chronic hypoxia (CH) is characterized by long-term hypoxia that is associated with microvessel proliferation and basal membrane (BM) degradation in tissues. The IL-6/JAK2/STAT3/MMP-9 pathway has been described in a variety of human cancers and plays an essential role in microvessel proliferation and BM degradation. Therefore, this study investigated the role of the IL-6/JAK2/STAT3/MMP-9 pathway in hypoxia-mediated microvessel proliferation and BM degradation in the rat bone marrow. Eighty pathogen-free Sprague Dawley male rats were randomly divided into four groups (20 per group)—control group, CH group (exposed to hypoxia in a hypobaric chamber at a simulated altitude of 5000 m for 28 d), CH + STAT3 inhibitor group (7.5 mg/kg/d), and CH + DMSO group. Microvessel density (MVD) and BM degradation in the bone marrow were determined by immunofluorescence staining and transmission electron microscopy. Serum IL-6 levels were assessed by enzyme-linked immunosorbent assay (ELISA), and the levels of P-JAK2, P-STAT3, and MMP-9 were assessed by western blot analysis and real-time reverse transcription PCR (RT-PCR). Hypoxia increased serum IL-6 levels, which in turn increased JAK2 and STAT3 phosphorylation, which subsequently upregulated MMP-9. Overexpression of MMP-9 significantly promoted the elevation of MVD and BM degradation. Inhibition of STAT3 using an inhibitor, SH-4-54, significantly downregulated MMP-9 expression and decreased MVD and BM degradation. Surprisingly, STAT3 inhibition also decreased serum IL-6 levels and JAK2 phosphorylation. Our results suggest that the IL-6/JAK2/STAT3/MMP-9 pathway might be related to CH-induced microvessel proliferation and BM degradation in the bone marrow.

## 1. Introduction

Chronic hypoxia (CH) is a condition where the partial pressure of oxygen in the blood is too low to saturate hemoglobin, which may be due to various reasons, including the decrease in the amount of breathable oxygen owing to a reduced barometric pressure [1], often encountered in pilots, mountain climbers, and people living at high altitudes. People inhabiting high altitudes are considered to be exposed to CH and show excessive erythrocytosis and hypoxemia [2, 3]. It is estimated that approximately 140 million people live permanently at altitudes above 2500 m above sea level. The prevalence of CH at high altitude is increased by 10% in Peruvian Andes at an altitude of about 2500 m and by 17.8% in Chinese Han men who migrated to the Qinghai–Tibetan plateau at an altitude of 3700–5000 m [2, 4, 5]. Previous studies showed that CH arises due to the excessive production of erythrocytes, which increases the blood viscosity causing blood flow retardation leading to exacerbated hypoxic-ischemic injury and eventually angiogenesis and basal membrane (BM) degradation of the blood vessel in tissues [6–10]. In addition, in a previous study, we showed that patients with CH frequently exhibit an erythematic facial color with marked congestion of the mucosa and conjunctiva as a result of the formation of new vessels and that CH is associated with changes in bone marrow MVD [6]. However, the mechanisms underlying the development of CH-induced changes in the microvessels are relatively unknown.

The Janus kinase/signal transducer and activator of transcription (JAK/STAT) pathway plays a vital role in mediating angiogenesis against ischemic injury [11–17]. Importantly, JAK2/STAT3 signaling has been specifically shown to protect against cerebral ischemia-reperfusion injury by inducing microvessel proliferation [18–23]. A previous study reported the role of IL-6/JAK2/STAT3 in nonsmall-cell lung cancer cell proliferation and migration through microvascular proliferation [21]. Other studies have validated this and have identified the pathways regulating the process [18–23]. Proinflammatory cytokine interleukin-6 (IL-6) released from inflammatory cells binds to IL-6 receptor *α* and active gp130 to induce the phosphorylation of JAK2 (P-JAK2), in response to hypoxia [24–27]. P-JAK2 promotes the overexpression of MMP-9 in the vascular endothelial cells, leading to angiogenesis and BM degradation by activating the phosphorylation of STAT3 tyrosine residues (P-STAT3) [7]. Interestingly, some studies have reported that IL-6 could regulate JAK2-STAT3 signaling during the metastasis of gastric cancer by regulating epithelial-mesenchymal proliferation and transition [23]. These results suggest that the IL-6/JAK2/STAT3 pathway is associated with microvascular proliferation.

MMPs are a family of zinc-dependent endopeptidases with more than 20 different members [28, 29]. MMP-9, a member of the MMP family, plays a crucial role in regulating angiogenesis and BM degradation under hypoxic conditions [30, 31]. Overexpression of MMP-9 is often observed in different malignant tumors and has been shown to promote metastasis and invasion by inducing angiogenesis and BM degradation [32–34]. It has been shown that the function of MMP-9 may be regulated by many upstream pathways, including the IL-6/JAK2/STAT3 pathway [33, 35–39]. Based on these, we hypothesized that the IL-6/JAK2/STAT3/MMP-9 pathway is associated with the microvascular proliferation and BM degradation induced by CH.

This study aimed to understand the mechanisms underlying hypoxia-induced microvascular proliferation and BM degradation in rat bone marrow, and to identify the role of the IL-6/JAK2/STAT3/MMP-9 pathway in the development of CH.

## 2. Materials and Methods

### 2.1. Animals

Specific pathogen-free (SPF) male Sprague Dawley (SD) rats weighing 200 ± 20 g were purchased from the Animal Center of Xi'an Jiaotong University, China. The experimental protocol was approved by the Institutional Animal Care and Use Committee of Qinghai University and was in compliance with the animal management rules of the Chinese Ministry of Health. All rats were housed at an ambient temperature of 18 ± 2°C and a relative humidity of 40–60% throughout the experiment and were fed a standard pellet diet and water ad libitum.

### 2.2. Reagents and Instrumentation

An STAT3 inhibitor (CAS no.14556632-40-8; product no. SH-4-54; purity >98%) was purchased from Med Chem Express Co., Ltd. (USA). Anti-MMP-9 (catalog no. # ab38898), Anti-P-STAT3 (catalog no. # ab32143), STAT3 (catalog no. # ab68153) Anti-P-JAK2 (catalog no. # ab32101), JAK2 (catalog no. # ab108596), and anti-β-actin (catalog no. # ab8229) antibodies were obtained from Abcam (Cambridge, MA, USA). The sequences of the forward and reverse primers for MMP-9 and GAPDH were designed using primer 3 and synthesized by Jinsirui Co., Ltd. (Nanjing, China). The IL-6 ELISA kit (catalog no. # EK0410) was purchased from Boster Biotechnology Co., Ltd. (Wuhan, China). The QIAGEN miRNeasy mini kit (catalog no. # 217004) was obtained from QIAGEN (Germany). The TaKaRa PrimeScript RT reagent kit (catalog no. #RR036A) and TaKaRa TB Green Premix Ex Taq (catalog no. #RR820A) were purchased from TaKaRa (Japan). ProLong™ Gold antifade reagent (P36931) was obtained from Invitrogen (Thermo Fisher Scientific, USA). The CMS rat model was established in an automatically adjusted low-pressure hypobaric chamber (DYC-300, Guizhou Fenglei Oxygen Chamber Co., Ltd., Guizhou, China).

### 2.3. Establishment of the Animal Model

Rats were randomly divided into four groups—control group, CH group, CH + inhibitor (STAT3) group, and CH + DMSO group (20 rats per group). The rats in the control group were kept in permanent normoxia, for 28 days. All rats except those in the control group were kept in a hypobaric chamber for 28 d [40–43] (24 h per day), where the pressure and oxygen concentration was the same as that at an altitude of 5000 m.

### 2.4. The Experimental Protocol

The recommended dosage of the STAT3 inhibitor for the mouse is 15 mg/kg/d, and the equivalent dose for rats is approximately 0.5 times the mouse dose which was dissolved in DMSO (1.6378 ml/10 mg) to a final concentration of 1.3% DMSO, and the mixture was administered [8]. Thus, rats in the CH + inhibitor group were treated with 7.5 mg/kg/d STAT3 inhibitor by oral gavage [40, 41] daily and those in the CH + DMSO group were treated with the same volume of 0.9% normal saline + DMSO daily. The rats were anesthetized using urethane (1.0 g/kg) and sacrificed by bleeding the abdominal aorta. Blood samples were collected for routine tests using a blood cell analyzer obtained from Mindray Biomedical Electronics Co., Ltd. (BC-5000Vet, Shenzhen, China), and Hb, HCT, and erythrocyte counts were recorded.

### 2.5. Collection of Bone Marrow Samples

The thigh bones of the rats were extracted, homogenized, and centrifuged in a centrifuge tube with 15 ml of 0.9% normal saline at 3000 rpm for 5 min, and filtered through a 300 mesh strainer for collecting the bone marrow. A part of the bone marrow sample was flash-frozen in liquid nitrogen and stored at −80°C for further extraction of RNA and proteins. The remaining samples were fixed in 4% paraformaldehyde and 2.5% glutaraldehyde for immunofluorescence staining and electron microscopy.

### 2.6. ELISA for Serum IL-6 Levels

In brief, 4 ml blood was collected from the abdominal vein in a serum separator tube and centrifuged at 3000 rpm for 15 min. The separated serum samples were stored at −80°C for subsequent cytokine analysis. Serum IL-6 levels were quantified by ELISA using EK0410 following the manufacturer's instructions.

### 2.7. Immunofluorescence Staining for MMP-9 and Microvessel Density (MVD)

Frozen sections were prepared for immunofluorescence staining using a LEICA CM1950 (LEICA CM1950, Germany) and an SP-HRP kit (SP-9000) purchased from ZSGB Biotechnology (ZSGB-BIO China Co., Ltd., Beijing, China). In brief, sections were washed three times (5 min each) with 0.01 mmol/L PBS (pH 7.4) and blocked with normal goat serum. Then, they were incubated at 4°C overnight with rabbit anti-rat anti-MMP-9 (1 : 600) in NCM universal antibody diluent (cat. no: WB100D) purchased from New Cell and Molecular Biotech (Suzhou, China), washed six times (5 min each) with PBS, and incubated with a biotinylated goat anti-rabbit antibody (cat. no: 111-545-003, Jackson, America) for 30 min at room temperature. After rinsing in PBS for 2 h, the sections were stained with ProLong TM Gold antifade reagent for 10 s. The reaction product was stored in the dark at room temperature and observed using a ZEISS LSM 880 (ZEISS LSM 880, Germany).

MVD was determined using immunofluorescence staining with MMP-9 as a marker for microvessel endothelium, as described previously [6]. Slides were examined at a magnification of ×400 to determine the highest vascular density in the bone marrow. Six fields were examined for each slide by two pathologists blinded to the study group, and the field with the highest MVD was selected for counting. The average of the six fields was recorded as the MVD for each rat. Four slides were observed for five rats each in each group.

### 2.8. Transmission Electron Microscopy

BM degradation in the bone marrow was examined by transmission electron microscopy (TEM). Tissues were fixed with 3% buffered glutaraldehyde and stored in a refrigerator overnight. After that, they were rinsed in 0.1 M phosphate buffer and postfixed for 2 h with 1% osmium tetroxide in 0.125 M sodium cacodylate buffer, dehydrated in increasing concentrations (30–100%) of ethanol, rinsed in acetone, and embedded in Araldite. Ultrathin sections (500 nm) were stained with uranyl acetate and lead citrate and examined using a Tecnai Spirit Bio-TWIN electron microscope (Czech).

### 2.9. Real-Time Quantitative PCR

The total RNA was extracted from the frozen bone marrow using QIAGEN miRNeasy mini kit and quantified using a NanoDrop according to the manufacturer's instructions. cDNA was synthesized using the TaKaRa PrimeScript RT reagent kit. The mRNA expression of MMP-9 was determined by TaKaRa TB Green Premix Ex Taq on an ABI 7500 Real-time PCR system (Bio-Rad, CA, USA). The primers used were as follows: MMP-9 (forward: 5′-GCATCTGTATGGTCGTGGCT-3′; reverse: 5′-TGCAGTGGGACACATAGTGG-3′) and GAPDH (forward: 5′-AGTGCCAGCCTCGTCTCATA-3′; reverse: 5′-GAACTTGCCGTGGGTAGAGT-3′). The relative gene expression was calculated using the 2^−ΔΔCt^ method, and all values were normalized to the gene expression level of the housekeeping gene GAPDH.

### 2.10. Western Blotting

The protein expression levels of MMP-9, P-JAK2, and P-STAT3 in the bone marrow were determined by western blot analysis. Proteins were isolated from frozen spleen and bone marrow tissues by homogenization in RIPA buffer containing 1 mmol/L PMSF and centrifuged at 11,000 g for 10 min at 4°C to collect the supernatant. The protein concentration was measured using the bicinchoninic acid assay with bovine serum albumin as a standard sample. Proteins were resolved with 10% SDS-PAGE and transferred to polyvinylidene difluoride membranes, which were blocked with 5% nonfat milk for 1 h and incubated with anti-MMP-9 (1 : 1000), anti-P-JAK2 (1 : 2000), anti-P-STAT3 (1 : 3000), and anti-*ß*-actin (1 : 300) antibodies at 4°C overnight. Then, the membranes were incubated with goat anti-mouse/anti-rabbit immunoglobulin *G* (1 : 20000) for 1 h and detected with an enhanced chemiluminescence kit (ECL, Biyuntian Biotech Institute).

### 2.11. Statistical Analyses

The results were analyzed using the SPSS 19.0 software (SPSS, Inc., Chicago, IL) and expressed as the mean ± SD (normal distribution). Differences among groups were analyzed by one-way analysis of variance (ANOVA), followed by the Student–Newman–Keuls test and Dunnett's multiple comparison test. A *P* value less than 0.05 was considered to be statistically significant.

## 3. Results

### 3.1. Characteristics of the Rat Model of Chronic Hypoxia

The CH rat model was established in the CH, CH + inhibitor, and CH + DMSO groups. The rats under CH showed typical symptoms, including cyanosis in the mucous membrane of the lips, tongue, ears, palms, and soles of feet compared to the control group. In addition, on day 28, the RBC, Hb, and HCT in the CH group increased to 11.93 ± 1.64 × 10^12^/L, 244.12 ± 26.38 g/L, and 68.82 ± 8.16%, respectively, compared to 8.18 ± 0.75 × 10^12^/L, 177.33 ± 17.95 g/L, and 44.74 ± 5.40%, respectively, in the control group (*P* < 0.05; Table 1). Treatment with an STAT3 inhibitor significantly decreased the levels of RBC, Hb, and HCT in the CH + inhibitor group (9.65 ± 1.13 × 10^12^/L, 205.41 ± 21.26 g/L, and 54.27 ± 6.71%) compared to those in the CH + DMSO group (11.96 ± 1.67 × 10^12^/L, 243.18 ± 28.61 g/L, and 68.10 ± 8.78%, respectively) (*P* < 0.05; Table 1). However, the RBC, Hb, and HCT levels in the CH + inhibitor group were significantly higher than those in the control group (*P* < 0.05; Table 1).

### 3.2. Serum IL-6 Levels Increased under Hypoxia

**Figure 1:**
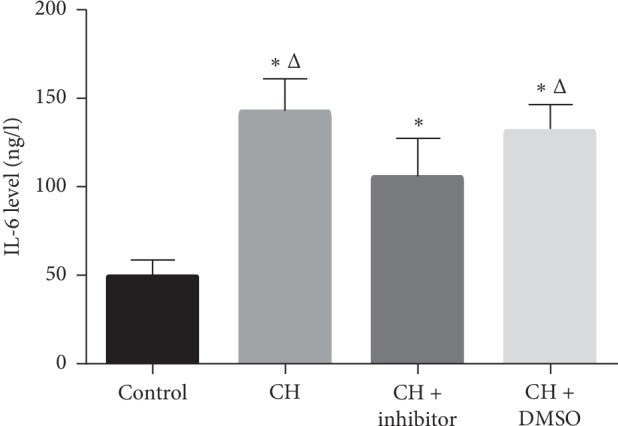
Serum IL-6 levels increased in CH rats. Control: control group; CH : chronic hypoxia group; CH + inhibitor group: chronic hypoxia + STAT3 inhibitor; and CH + DMSO group. Results are presented as mean ± SEM (*n* = 14 rats per group). ^Δ^*P* < 0.05 vs. CH + inhibitor; ^*∗*^*P* < 0.05 vs. control.

The serum IL-6 levels are shown in Figure 1. The serum IL-6 level in the CH group increased to 142.31 ± 18.75 ng/L from 48.91 ± 9.63 ng/L in the control rats (*P* < 0.05). Treatment with an STAT3 inhibitor decreased the serum Il-6 levels to 104.95 ± 22.52 ng/L from 142.31 ± 18.75 ng/L and 30.82 ± 15.74 ng/L, respectively, in the CH and CH + DMSO groups (*P* < 0.05). In addition, the IL-6 level in the CH + inhibitor group was significantly higher than that in the control group. However, there was no difference in the serum IL-6 levels between CH and CH + DMSO groups (*P* < 0.05).

### 3.3. JAK2/STAT3 Phosphorylation/Activation Increased with CH and Was Moderately Reversed with STAT3 Inhibitor Treatment

**Figure 2:**
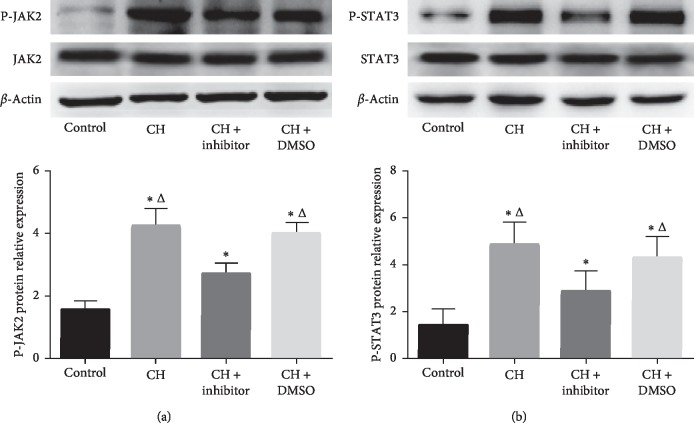
Phosphorylation of JAK2 and STAT3. Control: control group; CH : chronic hypoxia group; CH + inhibitor group: chronic hypoxia + STAT3 inhibitor; and CH + DMSO group. Results are presented as mean ± SEM (*n* = 6 rats per group). ^*∗*^*P* < 0.05 vs. control; ^Δ^*P* < 0.05 vs. CH + inhibitor.

Western blot analysis was performed to identify whether the IL-6/JAK2/STAT3/MMP-9 pathway was involved in microvessel proliferation. The results showed that phosphorylation levels of JAK2 and STAT3 were significantly increased in the hypoxia groups compared to those in the control group (*P* < 0.05; Figure 2). Treatment with an STAT3 inhibitor significantly decreased the phosphorylation of JAK2 and STAT3 (*P* < 0.05; Figures 2(a) and 2(b)). However, there were no differences in phosphorylation levels of JAK2 and STAT3 between the CH and CH + DMSO groups (*P* > 0.05; Figures 2(a) and 2(b)).

### 3.4. MMP-9 Was Upregulated in the Bone Marrow of CH Rats and Downregulated after STAT3 Inhibitor Treatment

**Figure 3:**
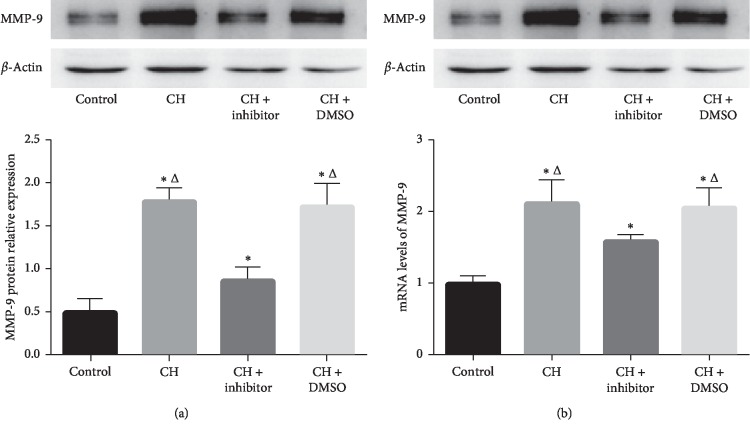
MMP-9 protein and mRNA expression increased in the bone marrow in the CH group and were decreased significantly with STAT3 inhibitor treatment. Control: control group; CH : chronic hypoxia group; CH + inhibitor group: chronic hypoxia + STAT3 inhibitor; and CH + DMSO group. Results are presented as mean ± SEM (*n* = 6 rats per group). ^*∗*^*P* < 0.05 vs. control; ^Δ^*P* < 0.05 vs. CH + inhibitor.

Western blot analysis showed that the MMP-9 levels in the hypoxia groups were higher than those in the control group (*P* < 0.05; Figure 3(a)). The STAT3 inhibitor decreased the expression of MMP-9 compared to the CH and CH + DMSO groups. MMP-9 expression in the CH + STAT3 inhibitor group was significantly higher than that in the control group (*P* < 0.05; Figure 3(a)).

In addition, the mRNA expression detected by using RT-PCR showed that MMP-9 expression was significantly increased in the hypoxia groups compared to that in the control group (*P* < 0.05; Figure 3(b)). The STAT3 inhibitor significantly downregulated MMP-9 gene expression compared to that in the CH and CH + DMSO groups. However, the MMP-9 mRNA level in the CH + inhibitor group was higher than that in the control group (*P* < 0.05; Figure 3(b)). MMP-9 expression in the CH group was similar to that in the CH + DMSO group (*P* < 0.05; Figure 3(b)).

### 3.5. CH Increased MVD in the Bone Marrow, and STAT3 Inhibitor Treatment Significantly Attenuated the CH-Induced MVD

**Figure 4:**
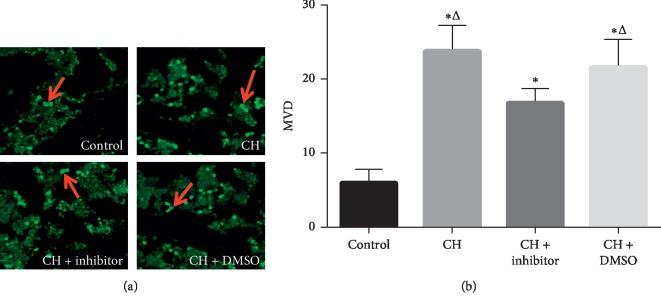
MVD was significantly increased in the bone marrow of the hypoxia groups compared to the control group. Treatment with an STAT3 inhibitor lowered the CH-induced MVD. Control: control group; CH: chronic hypoxia group; CH + inhibitor group: chronic hypoxia + STAT3 inhibitor; and CH + DMSO group. Results are presented as mean ± SEM (*n* = 6 rats per group). Red arrows indicate MMP-9-positive microvessels. ^*∗*^*P* < 0.05 vs. control; ^Δ^*P* < 0.05 vs. CH + inhibitor.

MVD in the bone marrow was determined by immunofluorescence (Figure 4), and MMP-9-positive vascular endothelial cells were identified by green staining. Immunofluorescence staining showed that MVD in the bone marrow was significantly higher in the hypoxia groups than that in the control group (*P* < 0.05; Figures 4(a) and 4(b)). The STAT3 inhibitor treatment significantly decreased the CH-induced MVD (*P* < 0.05; Figures 4(a) and 4(b)). However, the MVD levels in the CH + inhibitor group were higher than those in the control group (*P* < 0.05; Figures 4(a) and 4(b)). There was no difference between the CH group and the CH + DMSO group (*P* < 0.05; Figure 4(b)).

### 3.6. BM Degradation Occurred in the Bone Marrow of CH Rats and Was Attenuated by STAT3 Inhibitor

**Figure 5:**
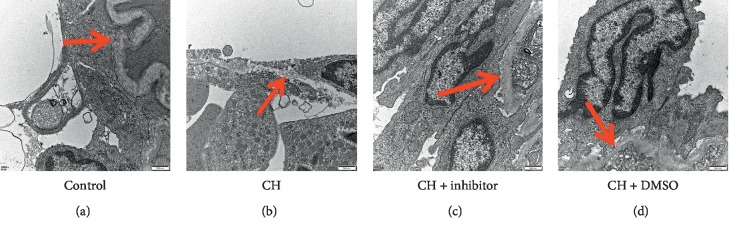
Ultrastructural analyses of BM. Photomicrographs of BM of one randomly selected slide per group. Scale bar = 500 nm. BM degradation was higher in the CH group compared to that in the control group. Treatment with an STAT3 inhibitor significantly decreased BM degradation. Control: control group; CH: chronic hypoxia group; CH + inhibitor group: chronic hypoxia + STAT3 inhibitor; and CH + DMSO group. Red arrows indicate the BM of microvessels. (a) Control. (b) CH. (c) CH + inhibitor. (d) CH + DMSO.

The BM of microvessels in the bone marrow was observed by TEM (Figure 5). The control group showed a thick and continuous BM, while in the CH group, the BM was uneven and thin with increased BM degradation (Figure 5). The BM thickness in the CH + inhibitor group was higher than that in the CH group (Figure 5). However, the CH + inhibitor group BM was uneven and degraded compared to the control group BM. There were no significant differences between the CH group and the CH + DMSO group (Figure 5).

## 4. Discussion

Our study has five major findings: (1) under chronic hypoxic conditions, the rat model developed erythropoiesis; (2) angiogenesis and BM degradation were the significant pathological changes under long-term hypoxia in the bone marrow microvessels; (3) during CH, an increase in serum IL-6 induced the upregulation of MMP-9 through the activation of the JAK2/STAT3 pathway; (4) the MMP-9 concentration in the bone marrow in CH rats was consistent with the analysis of MVD and BM degradation; (5) treatment with an STAT3 inhibitor attenuated the MMP-9 downregulation and hypoxia-induced pathological changes in the bone marrow microvessels.

Erythropoiesis occurs in response to hypoxia [42–45]. Sustained hypoxia exposure triggers subsequent vascular pathologic changes such as an increase in blood viscosity and angiogenesis leading to BM degradation [7, 8, 10, 38]. Consistent with previous studies, we observed an increased MVD in the hypoxic experimental groups [6–8]. It has been reported that upstream elements of the MMP-9 pathway are involved in the response to hypoxia [21, 35, 36, 38, 39]. In a previous study, we showed that hypoxia induced the development of MVD in young rats [6]. Therefore, in this study, we used adult rats to examine the role of the IL-6/JAK2/STAT3/MMP-9 pathway in the development of hypoxia-induced MVD.

Our results show that MMP-9 expression is enhanced in hypoxia. Consistent with previous studies [46, 47],we observed that increased MMP-9 expression results in an elevation of MVD and BM degradation [30–34]. In our study, we observed an increase in MMP-9 in the CH rat bone marrow, which resulted in an increased MVD and BM degradation. Interestingly, the increase in MVD and BM degradation appears to be dependent on MMP-9. Studies have shown that some cells, including neutrophils, macrophages, fibroblasts, and endothelial cells, can synthesize and secrete MMP-9 [47, 48]. MMP-9 plays a critical role in basement membrane degradation since the basement membrane contains collagens, including type IV collagen, which can be degraded by MMP-9 [30, 31]. Basement membrane destruction is an essential step during tumor development and supports tumor invasion and metastases by promoting angiogenesis. Consistent with these findings, we observed that the overexpression of MMP-9 increased the MVD and BM degradation [32–34]. MMP-9 is also expressed in vascular endothelial cells. Additionally, previous studies have shown that vascular endothelial cells are produced under hypoxic conditions, which enhance microvessel proliferation. Consistent with these findings, we observed that, under hypoxia, the MVD increased because the vascular endothelial cells are produced [46–50].

Studies have shown that the mechanism of microvessel proliferation and basal membrane degradation in other diseases, such as cancers and strokes, involves the JAK2/STAT3, IL-6/JAK2/STAT3, and MMP-9 pathways, which regulate the progression of tumor metastasis [9–11]. Currently, the pathways involved in hypoxia-mediated microvessel proliferation and basal membrane degradation have not been reported, except the IL-6/JAK2/STAT3/MMP-9 pathway in our study [37–39]. However, the mechanism underlying hypoxia-mediated microvessel proliferation and BM degradation has not been studied. To our knowledge, this is the first report on the involvement of the IL-6/JAK2/STAT3/MMP-9 pathway in the development of hypoxia-induced BM degradation.

**Figure 6:**
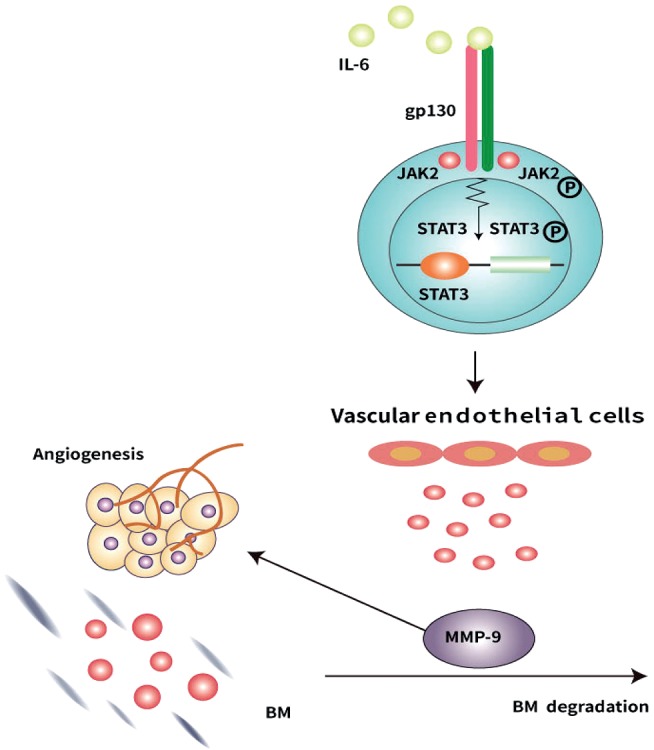
The role of the IL-6/JAK2/STAT3/MMP-9 pathway in the regulation of hypoxia-mediated microvessel proliferation and basal membrane degradation. Under hypoxic conditions, the increased IL-6 binds to IL-6 receptor *α* and activates gp130 to induce JAK2 phosphorylation. P-JAK2 upregulates MMP-9 secretion in the vascular endothelial cells leading to microvessel proliferation and basal membrane degradation.

In order to understand how the IL-6/JAK2/STAT3/MMP-9 pathway regulates the microvessels under hypoxia, the specific processes should be analyzed in detail. IL-6, one of the dominant inflammatory cytokines [24–27], markedly upregulates the phosphorylation of JAK2 by binding to IL-6 receptor *α* (IL-6R*α*) and activating gp130 [12]. P-JAK2 subsequently induces STAT3 phosphorylation. P-STAT3 induces the overexpression of MMP-9 in the vascular endothelial cells leading to microvascular proliferation and BM degradation [Figure 6]. Previous studies have reported a hypoxia-induced increase in IL-6 [26, 51], which was also observed in our study. Our results suggest that CH significantly increased serum IL-6 which induced the increased phosphorylation of JAK2 and STAT3 resulting in the overexpression of MMP-9. These results are in line with previous studies [18–29].

To further verify the involvement of the IL-6-JAK2-STAT3-MMP-9 pathway, we inhibited STAT3 using an inhibitor [45]. Treatment with the STAT3 inhibitor decreased STAT3 phosphorylation in the CH rat bone marrow and resulted in a decrease in tissue MMP-9 concentration [52–55]. With the STAT3 inhibitor, many factors should be analyzed. Firstly, it is well established that regulation of erythropoiesis and vasculogenesis is an adaption to hypoxia in the healthy body [6–9]. On the one hand, previous studies showed that hematopoietic and endothelial cell lineages share common progenitors that affect the proliferation and differentiation of bone marrow-derived or umbilical cord blood-derived endothelial progenitor cells, which in turn promote angiogenesis [56, 57]. On the other hand, it is well known that the bone marrow is characterized by the cellular diversity and complex functions [58].Importantly, vascular endothelial cells play a critical role in microvessels of the bone marrow [59, 60]. Some studies showed that STAT3 is expressed in vascular endothelial cells [19]. Therefore, after STAT3 inhibitor treatment, endothelial cell lineages and their common progenitor cells were inhibited resulting in suppression of microvessel proliferation. It has been shown that the BMSCs in the bone marrow were inhibited by the inhibition of JAK2/STAT3 signaling which is consistent with our results [59]. Additionally, our results showed that, under hypoxic condition, the microvessels from the vascular endothelial cells were enhanced by STAT3 overexpression. Interestingly, other studies have shown that, under hypoxia, bone marrow mesenchymal stem cell migration and osteogenic differentiation are enhanced via the STAT3 signaling pathway which is consistent with our results [60]. STAT3 inhibition also decreased serum IL-6 levels and JAK2 phosphorylation, along with a reduction in STAT3 phosphorylation. This may be due to the effect of a negative feedback mechanism. We speculate that, with STAT3 inhibition, the MMP-9 expression downregulated, which attenuated the hypoxia-induced increase in MVD and BM degradation. The decreased MMP-9 level subsequently downregulated the serum IL-6 level through a negative feedback mechanism, which in turn decreased JAK2 phosphorylation, which further decreased STAT3 activation [61–64].

We observed that the levels of RBC, Hb, and HCT were increased under CH, which is consistent with previous results [6]. However, these levels were reduced after treatment with the STAT3 inhibitor. There is one possible explanation for this result. A previous study showed that EPO activated the phosphorylation of JAK2 by binding to the EPO receptor [64, 65]. In addition, we have previously reported that the EPO level is upregulated under hypoxia [6]. In the current study, JAK2 phosphorylation was decreased in the CH + inhibitor group compared to that in the CH group. Therefore, it is possible that the decreased JAK2 might affect the binding of EPO to its receptor resulting in a decrease in functional EPO causing a decrease in RBC, Hb, and HCT [13].

In summary, during CH, an increase in serum IL-6 induced the upregulation of MMP-9 through the activation of the JAK2/STAT3 pathway. Treatment with an STAT3 inhibitor downregulated MMP-9 expression and attenuated the hypoxia-induced microvessel changes. Accordingly, our results suggest that the IL-6/JAK2/STAT3/MMP-9 pathway is associated with BM degradation and microvessel proliferation in the bone marrow of rats exposed to CH. However, our observation and report of the involvement of the IL-6/JAK2/STAT3/MMP-9 pathway in the regulation of hypoxia-mediated microvessel proliferation and basal membrane degradation is the first of its kind. For the limitation of our study, it even seemed that IL-6/JAK2/STAT3/MMP-9 pathway involved in hypoxia-mediated microvessels changes and further research is needed in future from different prospects.

In our study, we found that the RBC, Hb, and HCT decreased with STAT3 inhibition under CH. It may be a potential therapeutic intervention for people living at high altitudes suffering from excessive erythrocytosis and hypoxemia [2]. Further, the identification of the involvement of the IL-6/JAK2/STAT3/MMP-9 pathway in hypoxia-induced BM degradation and microvessel proliferation in bone marrow lays a foundation for further studies in the field and the possibility for the development of novel therapeutic strategies.

## 5. Conclusions

In this study, we investigated the expression of IL-6/JAK2/STAT3/MMP-9 pathway elements and the effect of STAT3 inhibitor on microvessel proliferation and BM degradation in the bone marrow in CH rats. Our results showed that serum IL-6 levels are increased under CH, which activated the JAK2-STAT3 signaling pathway, which in turn upregulated the MMP-9 levels resulting in increased MVD of higher and thinner BM in rats. However, the administration of an STAT3 inhibitor resulted in a significant decrease in STAT3 phosphorylation and MMP-9 expression and subsequently lowered MVD. Interestingly, STAT3 inhibition also resulted in a downregulation of IL-6 and a decrease in JAK2 phosphorylation, possibly through a negative feedback mechanism. These results suggest that the IL-6-JAK2-STAT3-MMP-9 pathway is associated with microvessel proliferation and BM degradation in the bone marrow in CH rats. Further studies are required to identify the detailed mechanism underlying microvessel proliferation and BM degradation under CH.

## Figures and Tables

**Table 1:** Characteristics of the rat CH model.

Index	Control (*n* = 9)	CH (*n* = 9)	CH + Inhibitor (*n* = 9)	CH + DMSO (*n* = 9)
RBC (×10^12^/L)	8.18 ± 0.75	11.93 ± 1.64^a^	9.65 ± 1.13^b^	11.96 ± 1.67^c^
Hb (g/l)	177.33 ± 17.95	244.12 ± 26.38^a^	205.41 ± 21.26^b^	243.18 ± 28.61^c^
HCT (%)	44.74 ± 5.40	68.82 ± 8.16^a^	54.27 ± 6.71^b^	68.10 ± 8.78^c^

The RBC (×10^12^/L), Hb (g/l), and HCT (%) of rats exposed 28 days to chronic hypoxia (CH), CH + STAT3 inhibitor (CH + inhibitor), and CH + DMSO. Results are expressed as mean ± SEM (*n* = 9 rats per group). a ≠ b ≠ c, a, c < 0.05, b > 0.05 vs. control. a, c < 0.05 vs. CH + inhibitor.

## Data Availability

The data used to support the findings of this study are available from the corresponding author upon request.
